# Diets for Dairy Cows with Different Proportions of Crude Protein Originating from Red Clover Silage versus Soybean Meal: Ruminal Degradation and Intestinal Digestibility of Amino Acids

**DOI:** 10.3390/ani11082177

**Published:** 2021-07-23

**Authors:** Edwin Westreicher-Kristen, Ralf Blank, Monika Paschke-Beese, Wiebke Kühl, Siegfried Wolffram, Cornelia C. Metges, Andreas Susenbeth

**Affiliations:** 1 Institute of Animal Nutrition and Physiology, Christian-Albrechts-Universität zu Kiel, 24118 Kiel, Germany; blank@aninut.uni-kiel.de (R.B.); paschke-beese@aninut.uni-kiel.de (M.P.-B.); kuehl@aninut.uni-kiel.de (W.K.); wolffram@aninut.uni-kiel.de (S.W.); susenbeth@aninut.uni-kiel.de (A.S.); 2 Schothorst Feed Research BV, P.O. Box 533, 8200 AM Lelystad, The Netherlands; 3 Institute of Nutritional Physiology “Oskar Kellner”, Leibniz Institute for Farm Animal Biology (FBN), 18196 Dummerstorf, Germany; metges@fbn-dummerstorf.de

**Keywords:** lysine, methionine, degradability, digestibility, legume forage

## Abstract

**Simple Summary:**

The purpose was to assess the effect of exchanging crude protein of soybean meal with red clover silage (RCS) in total mixed rations (TMR) on ruminal degradation and intestinal digestibility (ID) of essential amino acids (EAA). Increasing proportions of RCS in the TMR increased the ruminal degradation and reduced the ID of EAA. The ruminal degradation of EAA of the TMR followed that of CP, but the ID of EAA differed from that of CP at higher levels of RCS in the diet. In conclusion, increasing proportions of RCS in TMR reduced the extent of EAA flow into the small intestine, reduced the ID of EAA, and consequently the intestinal absorbable EAA.

**Abstract:**

The purpose was to assess the effect of exchanging crude protein (CP) of soybean meal (SBM) with red clover silage (RCS) in total mixed rations (TMR) on ruminal degradation and intestinal digestibility (ID) of essential amino acids (EAA). Four TMR and their individual feed components were studied. The TMR were composed of forage and concentrates (75:25), with proportions of RCS in TMR of 0.15, 0.30, 0.45, and 0.60 on a dry matter basis, resulting in diet groups RCS15, RCS30, RCS45, and RCS60, respectively. The ruminal degradation of EAA was determined using the nylon bag technique. For this, samples of TMR and their individual feed components were ruminally incubated for 16 h. The feed residues of TMR obtained after 16 h of incubation were used for the determination of ID of EAA using the mobile-bag technique. Increasing RCS and reducing SBM proportions linearly increased (*p* < 0.01) the in situ ruminal degradation of individual EAA from 75.5% to 83.5%. The degradation of EAA followed the trend of CP degradation among TMR, except for Cys that was lower (*p* < 0.05) than that of CP in RCS60 (79.7% vs. 86.3%). The degradation of EAA in individual feed ingredients not always corresponded to the degradation of CP and was feed dependent. Increasing the proportions of RCS in the TMR linearly reduced (*p* < 0.001) the ID of EAA (except for Ile) from 78.2% to 67.3%. However, the ID of EAA did not always reflect the ID of CP, and in general, the differences between the ID of CP and EAA increased as RCS increased in the TMR. The ID values of most of the EAA were similar (*p* > 0.05) to ID of CP in RCS15 and RCS30, while they mostly differed (*p* < 0.05) in RCS45 and RCS60, and being higher for EAA than CP (except for Cys that was lower than CP, *p* < 0.05). Similar trends were observed for intestinal absorbable AA, resulting in higher values (*p* < 0.05) of intestinal absorbable for all EAA than of CP in diet RCS60. In conclusion, increasing levels of RCS in TMR reduced the extent of EAA flow into the small intestine, the ID of EAA, and consequently the intestinal absorbable EAA. Therefore, accurate determination of metabolizable AA must be considered for optimal diet formulation when including high proportions of RCS in diets of high-producing dairy cows.

## 1. Introduction

In recent years, the interest in feeding cows with home-grown feeds like forage legumes has increased [1]. From the legume forages, red clover (*Trifolium pratense* L.; RC) may have a relevant role in silage (RCS) production because of its ability to fixate atmospheric nitrogen (N) [2]. This may reduce the dependence on industrial N-fertilizer [3] and the greenhouse gas and nitrate emissions to the environment when included as a part of crop rotation [4]. Moreover, RC has good feed value due to less-marked decline in quality with advancing maturity [3] and is a good alternative for dairy cows due to its relatively high content in crude protein (CP). The RCS was proven to supply higher amounts of ruminally undegraded feed CP (RUP) to the intestine compared to grass silage [2,5,6] and to alfalfa [7]. All these characteristics support the potential of RCS to reduce the necessity of using protein feeds like soybean meal (SBM) or rapeseed meal in dairy farming.

In a previous in situ study [8], replacing the CP from SBM with RCS in diets of dairy cows reduced the RUP supply and the intestinal digestibility (ID) of RUP. However, besides the RUP flow to the intestine, the amino acid (AA) profile of RUP and the intestinal digestibility (ID) of individual AA are additional central elements of the protein value of feeds because this designates the AA supply to the cows’ metabolism. It has often been presumed that AA composition of the RUP largely mirrors the AA profile of the feedstuff [9,10,11]. However, AA of the RUP sometimes does not equal the AA needs of the cow in relations of concentration and digestibility, which can be relevant if the RUP proportion of a feed is high and a specific AA is low [12]. Some studies have also demonstrated that ruminal degradation modifies the AA profile of the RUP [13,14,15,16]. Therefore, whether or not the AA composition of a diet is similar to its RUP fraction is of great relevance for the evaluation of the protein value of a feed. Similarly relevant is to know whether the digestibility of RUP is comparable or not to the digestibility of the individual AA. Consequently, in this experiment, the diets studied in the in situ trial of Westreicher-Kristen et al. [8] were additionally investigated to evaluate the effect of exchanging CP from SBM with RCS in the diet on (1) ruminal degradation of AA, and (2) ID of AA in dairy cows. It was hypothesized that increasing the proportion of RCS in the diet decreases the protein value in terms of digestibility and supply of AA.

## 2. Materials and Methods

### 2.1. Rations Studied

Four total mixed rations (TMR) and their individual ingredients (maize silage, RCS, SBM, ground wheat, and lupine seed) were obtained from a feeding experiment reported by Schulz et al. [1] and were identical with those of an in situ trial reported by Westreicher-Kristen et al. [8] aimed at evaluating the ruminal degradation and ID of CP. The TMR were formulated to be iso-nitrogenous and to comprise similar forage to concentrate ratio (0.75:0.25). In the TMR, maize silage (MS) was replaced by target levels of RCS with increasing dry matter (DM) proportions of 0.15 (diet RCS15), 0.30 (diet RCS30), 0.45 (diet RCS45), and 0.60 (diet RCS60). The feed concentrates were SBM, wheat, and lupine seed (Table 1). The share of lupine seed was similar among TMR (average 87.8 g/kg DM). The RCS15 contained 159 g SBM/kg DM and was free of wheat, whereas RCS60 was free of SBM and was composed of 168 g wheat/kg DM. The chemical compositions of the four TMR and their individual feed components are shown in Table 2.

### 2.2. Ruminal Degradation of Amino Acids

The ruminal degradation of AA of the four TMR and of their individual feed ingredients was determined using the nylon bag technique. For this, four heifers (two German Black Pied and two Jersey × German Black Pied) with an average BW of 565 ± 29 kg and fitted with a rumen cannula (#2C, 10 cm i.d.; Bar Diamond Inc., Parma, ID, USA) were used. The animals received twice daily a diet composed of 3 kg of grass hay and 3 kg of concentrate (on DM basis) divided into two meals (07:00 and 15:00 h, 75 g of mineral-vitamin-premix once daily) and water for ad libitum consumption. The concentrate was composed (on fresh matter basis, g/kg) of 232 rye, 210 rapeseed meal, 185 corn gluten meal, 150 barley, 45 beet pulp, 40 maize, 20 rye bran, and 11 calcium carbonate. For the ruminal incubations, feed samples were ground through a 2.0-mm sieve (Retsch ZM1; Retsch GmbH, Haan, Germany) and 4.95 ± 0.04 g (on DM basis) was placed into nylon bags (10 × 20 cm, ~53 ± 10 µm of pore size, 24.7 mg DM sample/cm^2^ nylon bag, Type BG1020, Bar Diamond Inc., ID, USA). Ten nylon bags per sample and heifer were ruminally incubated for 16 h in three runs performed in three consecutive days (4, 3, and 3 bags/d per sample and heifer, respectively). The bags were deposited into the ventral sac of the rumen immediately after each morning feeding. After incubation, the bags were retrieved from the rumen, submerged in ice-cold water to reduce microbial activity, and immediately washed with cold tap water to remove the ruminal contents. Finally, all bags were freeze-dried and stored until analysis.

### 2.3. Intestinal Digestibility of Amino Acids

The feed residues obtained after ruminal incubation during 16 h were pooled per TMR to determine the ID of AA using the mobile-bag technique (MBT). For this, approximately 500 mg (on fresh matter basis) of feed residues were weighed into nylon bags (5 × 5 cm, ~53 ± 10 µm of pore size, Type BG505, Bar Diamond Inc., ID, USA). Ten nylon bags per TMR were used. The bags containing the feed residues were first pre-incubated in a pepsin-HCl solution to mimic abomasal digestion as described in detail by Westreicher-Kristen et al. (2018). For the small intestine incubation, three lactating cows (German Holstein) fitted with duodenal cannula were used as described by Westreicher-Kristen et al. [8]. Succinctly, the bags were placed randomly into the duodenum of the cows starting immediately after morning milking at approximately 06:00 h. A maximum of 12 bags/cow/d were introduced every 30 min. The intestinal incubation was performed in three consecutive runs (*n* = 3). To recover the bags, fecal material was searched for the bags beginning 8 h after first duodenal application, and once recovered, the bags were immediately washed with cold tap water and stored frozen (−20 °C) until analyses.

### 2.4. Chemical Analysis

The proximal constituents and fiber fractions of feeds were determined according to the official analytical methods in Germany [19] as described in Westreicher-Kristen et al. [20]. The N contents were determined using the Dumas procedure (method 4.1.2; [19]; TruSpec^®^N analyzer, LECO Corporation, St. Joseph, MI, USA), and the CP concentration was calculated by multiplying N concentration with a factor of 6.25.

For the AA analyses of TMR and individual ingredients, 0.1 g of each ruminally and duodenally incubated residue was weighed into a screw-capped test tube and mixed with 3 mL of 6N HCl. The tubes were purged with N and then hydrolyzed in an oven at 110 °C for 24 h. The hydrolyzed samples were mixed with the internal standard (DL-amino-n-butyric acid) and centrifuged at 1110× *g* for 15 min at 4 °C. The supernatant of the sample was analyzed according to Jones and Gilligan [21] using a LC-2000 Plus HPLC system (JASCO Deutschland GmbH, Pfungstadt, Germany) with a reversed-phase column (SUPELCOSIL™ LC-18, 3 µm, L × i.d. 15 cm × 4.6 mm; Sigma-Aldrich Chemie GmbH, Taufkirchen, Germany) protected by a guard column (SUPELCOSIL™ LC-18 Supelguard™ Cartridge 5 μm particle size, L × i.d. 2 cm × 4 mm), and a fluorescence detector (JASCO FP 2020 PLUS, JASCO Deutschland GmbH, Pfungstadt, Germany). The AA were derivatized using an o-phthaldialdehyde reagent solution. The mobile phase consisted of two solvents (A and B) with a flow rate of 1.1 mL/min. The solvent A contained 0.1 M sodium acetate (pH 7.2), methanol, and tetrahydrofuran in a ratio of 18:1. The solvent B was pure methanol. The peaks were recorded and integrated using the ChromPass/Galaxie Chromatography Data System version 1.10.1.2006 (JASCO Deutschland GmbH, Pfungstadt, Germany). Methionine was determined as methionine sulfone and cysteine as cysteic acid after oxidation with 98% performic acid according to Varzaru et al. [22]. For this, the oxidized samples were dried in a rotary evaporator, hydrolyzed, and analyzed after pre-column derivatization with o-phtaldialdehyde.

The CP and AA of the TMR diets, individual ingredients of the TMR, feed residues after ruminal incubation (TMR and their individual components), and of feed residues recovered after duodenal application (only for TMR) were analyzed in duplicate. Samples of TMR and their individual components were ground through a 0.5-mm sieve (Retsch ZM1; Retsch GmbH, Haan, Germany) before analysis, whereas samples of ruminal and intestinal feed residues were ground using a mortar and pestle.

### 2.5. Calculations and Statistical Analysis

The ruminal degradation of AA of the TMR and their individual components after 16 h ruminal incubation were calculated as the difference between the amount of feed in the bags before and after ruminal incubation. The ID of AA was calculated as the difference between AA in the residue after incubation in the rumen and the AA remaining after incubation in the small intestine. The intestinally absorbable AA (IAAA) was calculated as AA in RUP × ID of AA/100, where IAAA and AA in RUP are in g/kg AA, and ID of AA in percent. The total digestible AA (TDAA) was calculated as 1000–AA in RUP–IAAA, where TDAA, AA in RUP, and IAAA are in g/kg AA. The milk protein score (MPS) of TMR diets and individual feed ingredients and their feed residues after ruminal incubation was also calculated as (g of essential AA/kg CP)/(g essential AA in milk/kg of milk protein) according to Schingoethe [17], and the content of AA in the milk protein was according to Waghorn and Baldwin [18].

All statistical analyses were performed using the software package SAS (version 9.2, SAS Institute Inc., Cary, NC, USA). Data were subjected to ANOVA using the GLM procedure to examine the effect of TMR. The differences between least square means were assessed using Tukey’s test. The orthogonal polynomial contrasts were tested using the CONTRAST statement to examine the linear and quadratic effect of RCS proportions on the response variables. The significant differences were stated at *p* < 0.05 and the tendencies at *p*-values between 0.05 and 0.10.

## 3. Results

### 3.1. Amino Acid Concentration

The AA content, the MPS, and the ranking of the three most limiting AA relative to milk protein of TMR diets and their individual components are presented in Table 2. The highest concentration of total AA (TAA, in g/16 g N) was for SBM and lupine seed (92.8 and 92.4, respectively), followed by wheat (81.0), MS (71.4) and RCS (66.5). The MPS of individual TMR components varied between 0.438 (wheat) and 0.557 (SBM). According to MPS, Lys was the first limiting AA for all individual TMR components; Ile was the second limiting AA for MS, SBM, wheat, and lupine seed, and Met for RCS. Methionine was the third limiting AA for MS, SBM, and wheat, Ile for RCS, and Val for lupine seed. For the TMR diets, the concentration of TAA was reduced from 77.1 to 68.1 g/16 g N when increasing the proportion of RCS in the TMR from 0.15 to 0.60. The content of Lys reduced from 4.19 to 3.70 g/16 g N, and Met reduced from 1.45% to 1.16 g/16 g N when increasing the level of RCS from 15 to 60% of diet DM. The MPS of the TMR diets was also reduced from 0.474 to 0.427. Based on MPS, Lys and Ile were the first and second limiting AA for all TMR, respectively, whereas Met was the third limiting AA for RCS15 and RCS30, and Val for RCS45 and RCS60.

The AA content, the MPS, and the ranking of the three most limiting AAs relative to milk protein of the feed residues after 16 h of ruminal incubation of TMR and their individual components are shown in Table 3. The highest concentration of TAA in the feed residues was for wheat and SBM (84.5 and 81.0 g/16 g N) and the lowest for RCS (68.7 g/16 g N). Based on the MPS, Lys and Ile were the first and second most limiting AAs along all TMR diets and their individual ingredients. Methionine was the third limiting AA for RCS and wheat, and Val for MS, SBM, and lupine seed. Valine was the third most limiting AA for all TMR. The concentration of TAA in the feed residues of the TMR varied between 70.6 and 72.8 g/16 g N.

### 3.2. Ruminal Degradation of Amino Acids

The ruminal degradation of AA determined after 16 h of ruminal incubation for individual components and TMR are shown in Table 4. Increasing RCS and reducing SBM proportions augmented (*p* < 0.01) the degradation of all individual EAA in a linear fashion from 75.5% to 83.5%. Similarly, the degradation of all individual non-EAA (NEAA) increased linearly (*p* ≤ 0.02). Ruminal degradation of all individual EAA was similar to the ruminal degradation of CP along all RCS-diets (*p* > 0.05), except for Cys, which was lower than that of CP in RCS60 (79.7% vs. 86.3%, *p* < 0.05). Among the individual components of TMR, MS had a lower CP degradation than SBM (64.6% vs. 76.1%), followed by RCS (82.7%), while lupine seed and wheat were similar (92.7% and 94.2%, respectively). In general, the degradation of individual EAA of the individual TMR components reflected the degradation of CP, with some exceptions. For MS, degradation values of Cys, His, Lys, and Thr were lower (*p* < 0.05; 61.1%, 55.0%, 26.5%, and 47.3%, respectively), whereas the degradation of Leu (72.7%) was higher (*p* < 0.05) than the degradation of CP (64.6%). For RCS, only ruminal degradation of Cys (75.0%) deviated the most (*p* < 0.05) from CP degradation (82.7%). For SBM, ruminal degradation of Cys, His, Lys, Met, and Phe (86.1%, 81.4%, 80.5%, 80.8% and 78.9%, respectively) were higher (*p* < 0.05) than the degradation of CP (76.1%). For wheat, ruminal degradation of Ile, Met, and Thr (86.4%, 90.4%, and 91.4%, respectively) were lower (*p* < 0.05) than the degradation of CP (94.2%). For lupine, only ruminal degradation of Ile (86.3%) differed (*p* < 0.05) from CP degradation.

### 3.3. Intestinal Digestibility of Amino Acids

The ID of individual AA of the four TMR diets containing different levels of RCS and determined with the MBT is shown in Table 5. The ID of individual EAA decreased linearly (*p* < 0.001) with the exception of Ile that showed a quadratic response (*p* < 0.001). The ID of the EAA decreased on average from 78.2% to 67.3% when increasing the RCS proportion from 0.15 to 0.60 in the TMR. However, the ID of EAA did not always reflect the ID of CP, and in general, the differences between the ID of CP and EAA increased as RCS increased in the TMR. The ID values of most of the EAA were similar (*p* > 0.05) to the ID of CP in RCS15 and RCS30, while the ID values of most of the EAA differed (*p* < 0.05) from the ID of CP in RCS45 and RCS60. The intestinal absorbable CP (IACP) was reduced linearly (*p* < 0.001) from 18.4% to 7.88% when increasing levels of inclusion of RCS in the TMR diets. For the EAA, the IAAA was reduced linearly (*p* < 0.001, for all) on average from 19.2% to 11.0%. For RCS15, most of the IAAA values were similar to IACP, while for RCS60, all IAAA values were higher (*p* < 0.05) than that of IACP. Neither a linear nor quadratic effect of RCS on total digestible of Lys, Met, Phe, Thr, and Val was observed, but the total digestible Cys, His, Ile, and Leu decreased linearly (*p* ≤ 0.03 for all) when increasing RCS proportion in the TMR diets. In general, the total digestible EAA followed that of CP, averaged 94.8%, and differences between TMR diets were small in absolute terms.

## 4. Discussion

### 4.1. Amino Acid Composition of Individual Feeds and Total Mixed Rations

The TMR were formulated to study the effect of exchanging CP from SBM with CP from RCS. Simultaneously, the levels of CP and forage to concentrate ratio was maintained similar among TMR diets. Although the CP was similar between TMR diets (172–175 g/kg DM), increasing the RCS proportion in the diets resulted in a reduced content of all EAA (except Thr and Val). The latter resulted in a reduction of the total EAA by 10%. The content of Lys was reduced from 4.19 to 3.70 g/16 g N, and that of Met was reduced from 1.45 to 1.16 g/16 g N when increasing the proportion of RCS in the TMR from 0.15 to 0.60. Legume forages like RCS contain a considerable concentration of NPN. According to Schulz et al. [1], the RCS used in the present experiment contained 47.8% of NPN (% of total N), while lupine, SBM, and wheat contained 6.91%, 3.70%, and 17.7%, respectively. Non-protein N is composed of free AAs, peptides, nucleic acids, amides, amines, and ammonia [23], and its composition depends on the type of feed. Therefore, the reduction in content of the individual (especially Lys and Met) and total EAA can be primarily explained by the increased proportion of RCS in the diets. As a consequence, the MPS of RCS60 was the lowest among TMR diets. Lysine and Met have been recognized commonly as first-limiting EAA in the MP of dairy cows and the sequence of Lys and Met limitation is determined by their proportion in RUP [23]. Based on the MPS, Lys was identified as the first limiting AA in all RCS containing diets, followed by Ile as a second limiting AA.

The content of individual EAA of the TMR ingredients (except for RCS) were slightly different compared to table values published by the NRC [23] and CVB [24], and to the values of the Cornell Net and Carbohydrate Protein System (CNCPS) v6.5.5.1 [25,26], but differences were small in absolute terms. For SBM, the content of Lys was 5.90 g/16 g N and lower than NRC, CVB, and CNCPS values (6.28, 6.20, and 6.11 g/16 g N, respectively). The content of Met in SBM was 1.60 g/16 g N and was higher than the table values of NRC, CVB, and CNCPS (1.45, 140, and 1.34 g/16 g N, respectively). The feed tables of the NRC, CVB or CNCPS do not show AA contents for RCS.

### 4.2. Ruminal Degradation of Amino Acids

The ruminal degradation of dietary feed CP is a relevant factor affecting the protein flow to the small intestine of cattle. Hence, the ruminal degradation of AA and the AA composition of RUP are important factors to predict the amount of the individual AA that flows to the small intestine. The accurate measurement of the AA profile of RUP is expensive, laborious, and time consuming. This has maintained the use of the original AA profile to estimate duodenal supply [16]. In line with this, the Dutch protein evaluation system (DVE/OEB 2010; [27]) assumes that AA in forages and concentrate ingredients follow the same pattern of degradation as protein, and similarly the NRC [23] and the NorFor system [28] assumed that the AA composition of the RUP portion is similar to the original feedstuff. However, Edmunds et al. [16] found that the degradation of individual AA in forages can deviate from protein, while van Straalen et al. [15] and MacGregor et al. [13] concluded that the AA profile of concentrate feed and forage residue after ruminal incubation is more representative than that of the feedstuff. Therefore, an in situ trial was run in this study to assess the ruminal degradation of AA of RCS-diets and of their individual feed ingredients. In general, the ruminal degradation of individual EAA and NEAA of all TMR diets reflected the ruminal degradation of CP. However, a crucial question was whether this is also valid for the EAA of the individual feed ingredients used for the TMR. In the present study, most of the ruminal degradation values of EAA of lupine and RCS seeds were similar to the degradation values of the CP. For wheat and SBM, the ruminal degradation values of three and five EAAs were lower and higher than the degradation of CP, respectively. For MS, most of the degradation values of EAA differed from that of CP. The latter finding indicates that the degradation of EAAs is not always equal to the degradation of CP and depends on the type of feed. For MS, the ruminal degradation of CP was 64.6% and lower than the value reported by van Straalen et al. [15] of 74% after 12 h of ruminal incubation. The ruminal degradation values of all AA in MS varied between 26.5% (Lys) and 83.6% (Tyr). The degradation of Arg (38%) was the second lowest among all AA. Similarly, van Straalen et al. [15] reported values between 40% (Lys) and 81% (Ala) for MS. This high variation in the ruminal degradation of AA compared to CP can be attributed to microbial contamination, which could have a large effect because of the low CP content in MS. The low degradation and high content of Lys and Arg in the feed residues observed in this study can be an indication that the residues of MS contained microbial protein [15]. It is well known that microbial contamination may distort the estimation of ruminal incubation of CP [29] as well as of AA. In the present study, correction for microbial contamination was not considered. However, bags were strongly washed with cold water until the water became clear and a significant microbial contamination of the feed residues can be neglected. In general, the present study showed that the AA profile of feed ingredients studied changes during ruminal degradation and that AA profiles differed among feeds. However, these differences were less in the total diets possibly due to a compensation by the combination of different feeds. This also led to the conclusion that the general assumption that AA and CP have a similar degradation would not dramatically affect the total diets level, but differences in AA profile based on such an assumption would unfairly evaluate or rank individual feed in terms of AA supply capacity.

### 4.3. Intestinal Digestibility of Amino Acids

Based on the ruminal degradation values at the highest RCS level in the TMR, the ruminal degradation of all individual AA was numerically lower than that of CP. This could indicate a slightly improved quality of RUP in terms of AA profile for the RCS60 diet. However, an improved AA profile of RUP alone does not necessarily assure a simultaneously improved AA absorption and thus will not necessarily translate into improved animal performance. In this context, AA composition of RUP in combination with the ID of individual AA are factors that determine the amount of AA available from the feed for the animal. The ID of RUP decreased with increasing RCS proportion in the TMR and with increasing ruminal degradation of CP (or decreasing percentage of RUP). The latter indicates that the smaller the amount of CP that remains after ruminal degradation, the more resistant it is to intestinal digestion [8]. For EAA, the ID also decreased for all AA (except Ile) when increasing the RCS proportion in the TMR. However, the ID of EAA did not reflect the ID of CP and it seems that it was dependent upon the level of RCS in the TMR, and therefore, differences increased with increasing levels of RCS in the TMR. Whereas the ID of CP was reduced by 19% points when increasing the proportion of RCS in the TMR from 0.15 to 0.60, the ID of EAA was only reduced by 11% points on average. Moreover, while ID of CP and the majority of EAA were similar in RCS15 and RCS30, the ID of most of the EAA was different to the ID of CP in RCS45 and RCS60. The latter observation supports the idea that the ID of AA seems to be primarily feed dependent.

The reduction in ID of RUP was because exchanging the CP from SBM by RCS, which drastically shifted the origin of RUP from SBM to RCS [8]. Normally, protein rich feeds like SBM are characterized by higher RUP digestibility than that of forages [30]. The NRC [23] uses an ID of RUP of 93% for SBM and 65% for “all samples” of silage legume forages. The Dutch system (CVB Feed Table, [24]) uses an ID of RUP of 98% for SBM and 63% for RCS. Additionally, the ID of most of the EAA was higher than the ID of CP (except Cys that was mostly lower) in all TMR. Similar findings were reported by van Straalen [15] for different forage and concentrate feedstuffs using the MBT. Differences in ID between CP and AA can be due to the fact that the NPN fraction in RUP is mainly linked to components that are indigestible (e.g., cell walls) in the intestine [15]. Nitrogen bound to the ADF (ADiN) is a measure of indigestible N [31]. The RCS and SBM used in the TMR corresponded to 4.20% and 2.40% of total N, respectively. Consequently, the ADiN content (in % of total N) increased from 2.45 to 3.81 when increasing the RCS in the diets from 0.15 to 0.60 [1]. Moreover, the lignin and ADF content in the TMR increased by 46% and 15% when increasing the proportion of RCS, respectively. The increase in both chemical constituents in the TMR was mainly caused by the inclusion of RCS. The RCS had two-fold higher content of lignin than MS and six-fold than SBM, while the ADF content of RCS was slightly higher than MS and 2.6-fold higher than SBM. All the mentioned factors explain the reduction in digestibility of RUP and the increased differences between RUP and AA digestibility when increasing the proportion of RCS in the TMR.

The IAAA accounts for the proportion of AA that is available for absorption in the small intestine and is estimated considering the AA content in the RUP and the ID of the AA. The IAAA decreased with an increasing level of RCS proportion in the diets and reflected the IACP among all TMR diets. However, the IA of the EAA was higher than IACP in all TMR diets and differences increased with increasing proportion of RCS. The latter resulted in much higher IAAA values than IACP, especially in RCS60 diets for all EAAs. This occurred as a combination of relatively increased AA content in RUP and especially higher AA digestibility than CP in the RCS60 diet. Interestingly, the IA value for Lys was the highest among EAA, followed by Met for all TMR. No linear effect for TDCP and slight differences between TMR, but the TD of Cys, His, and Leu reduced linearly while the TD of Ile increased linearly. However, differences in TDAA between TMR diets were small in absolute terms. Thus, this study shows that increasing the proportion of RCS in the TMR reduces the AA supply to the cow for milk protein synthesis. This is in accordance with the results of the feeding experiment [1] where the same diets here evaluated were fed to milking cows, resulting in a decrease in milk protein content from 3.20% to 3.01% when increasing the proportion of RCS in the TMR from 0.15 to 0.60.

## 5. Conclusions

This study showed that increasing the proportions of RCS in TMR for cows reduced the extent of EAA flow into the small intestine, the ID of EAA, and consequently the intestinal absorbable EAA. The latter can result under in vivo conditions in reduced milk protein content and protein yield. However, under conditions of the present experiment, it seems that the maximum inclusion level of RCS amounts to 30% in the total diet without a substantial reduction in supply and ID of AA. At higher inclusion rates of RCS, it is recommendable to fine-tune and balance diets based on the supply of metabolizable AA.

## Figures and Tables

**Table 1:** Feed ingredients of the total mixed ratios (TMR).

Ingredient (g/kg DM)	TMR ^1^			
	RCS15	RCS30	RCS45	RCS60
Maize silage	610	466	316	162
Red clover silage	136	275	421	571
Soybean meal	159	108	55.0	-
Wheat	-	54.9	110	168
Lupine seed	85.9	87.0	88.8	89.6
Premix ^2^	9.10	9.10	9.20	9.40

^1^ The TMR was composed of forage and concentrates (0.75:0.25) with targeted proportions of red clover silage (RCS) in the TMR of 0.15 (RCS15), 0.30 (RCS30), 0.45 (RCS45), and 0.60 (RCS60) on a DM basis. ^2^ Premix contained salt, minerals, and vitamins.

**Table 2:** Chemical composition and concentration of amino acids (AAs) of individual feeds and total mixed rations (TMR).

Item	TMR Components ^1^					TMR ^2^			
	MS	RCS	SBM	W	LS	RCS15	RCS30	RCS45	RCS60
Chemical composition (g/kg DM)									
Organic matter ^3^	966	882	928	983	960	940	930	916	907
Crude protein	71.2	194	457	115	351	172	174	173	175
Ether extract	26.1	22.4	19.2	18.1	55.1	21.4	21.3	20.5	20.2
ANDFom ^4^	379	369	174	145	249	340	332	342	341
ADFom ^5^	219	296	113	32.1	180	212	219	239	244
Lignin(sa)	26.4	57.9	8.92	7.40	6.11	28.0	32.4	36.9	41.0
EAA ^6^ (g/16 g N)									
Cys	1.11	0.44	1.41	2.22	1.29	1.07	0.99	0.78	0.64
His	1.25	1.49	2.57	2.14	2.83	1.87	1.82	1.69	1.58
Ile	3.66 ^†^	3.86 ^‡^	4.57 ^†^	3.35 ^†^	4.26 ^†^	4.14 ^†^	4.22 ^†^	4.18 ^†^	3.89 ^†^
Leu	9.96	6.38	7.75	6.50	7.19	7.27	7.15	6.80	6.17
Lys	2.15 *	3.93 *	5.90 *	2.59 *	4.76 *	4.19 *	4.20 *	3.96 *	3.70 *
Met	1.80 ^‡^	1.29 ^†^	1.60 ^‡^	1.70 ^‡^	0.73	1.45 ^‡^	1.40 ^‡^	1.35	1.16
Phe	4.29	3.97	5.19	4.37	4.02	4.31	4.31	4.26	3.93
Thr	2.80	3.68	4.07	2.77	3.73	3.33	3.40	3.54	3.37
Val	4.85	4.79	4.71	4.07	3.95 ^‡^	4.51	4.70	4.80 ^‡^	4.56 ^‡^
TEAA	31.9	29.8	37.8	29.7	32.8	32.1	32.2	31.4	29.0
MPS ^7^	0.470	0.440	0.557	0.438	0.483	0.474	0.474	0.462	0.427
NEAA ^8^ (g/16 g N)									
Ala	8.82	4.96	4.48	3.63	3.74	5.32	5.27	5.06	4.62
Arg	1.73	2.31	7.01	3.88	10.4	4.89	4.48	3.95	3.35
Asp	4.92	10.4	11.8	5.11	10.4	8.61	9.05	9.33	9.01
Glu	10.9	7.04	18.1	27.5	21.7	13.5	12.9	11.6	10.3
Gly	3.93	4.16	5.11	4.08	4.86	4.43	4.43	4.24	3.92
Ser	3.06	3.69	5.24	4.58	5.29	3.94	3.92	3.85	3.60
Tyr	6.15	4.12	3.32	2.50	3.26	4.30	4.54	4.56	4.33
TNEAA	39.5	36.7	55.1	51.3	59.7	45.0	44.6	42.6	39.1
TAA	71.4	66.5	92.8	81.0	92.4	77.1	76.8	74.0	68.1

^1^ MS, Maize silage; RCS, Red clover silage; SBM, Soybean meal; W, Wheat; LS, Lupine seed. ^2^ The TMR was composed of forage and concentrates (0.75:0.25), with targeted ratios of red clover silage (RCS) in TMR of 0.15 (RCS15), 0.30 (RCS30), 0.45 (RCS45), and 0.60 (RCS60) on a DM basis. ^3^ Organic matter calculated as 1000–CA, where CA is in g/kg DM. ^4^ aNDFom, Neutral detergent fiber, α-amylase pre-treated, ash free. ^5^ ADFom, Acid detergent fiber, ash free. ^6^ EAA, Essential AA. ^7^ MPS, Milk protein score calculated as MPS = (g of EAA/kg crude protein)/(g EAA in milk/kg of milk protein), according to Schingoethe [17]. ^8^ NEAA, Non-essential AA. *^,†,^^‡^ Designate the order of the first, second, and third most limiting AA, respectively, within TMR components or TMR; based on estimations of the AA content of milk protein [18].

**Table 3:** Concentration of amino acids (AAs) of feed residues of individual feeds and total mixed rations (TMR) after 16 h of ruminal incubation.

Item	TMR Components ^1^					TMR ^2^			
	MS	RCS	SBM	W	LS	RCS15	RCS30	RCS45	RCS60
EAA ^3^ (g/16 g N)									
Cys	1.52	0.69	0.86	0.03	0.76	1.17	1.12	0.99	0.95
His	1.60	1.59	2.10	2.06	1.85	1.71	1.69	1.73	1.71
Ile	4.17 ^†^	4.09 ^†^	4.56 ^†^	8.02 ^†^	7.01 ^†^	4.02 ^†^	4.10 ^†^	4.25 ^†^	4.32 ^†^
Leu	8.04	6.67	7.60	4.65	4.35	6.78	6.87	6.99	6.97
Lys	4.68 *	4.82 *	5.05 *	4.39 *	4.60 *	4.81 *	4.75 *	4.99 *	4.81 *
Met	2.22	1.44 ^‡^	1.35	0.13 ^‡^	0.77	1.45	1.46	1.50	1.45
Phe	4.38	4.40	4.79	4.89	4.19	4.16	4.25	4.47	4.53
Thr	4.37	4.01	3.87	4.21	3.65	3.64	3.77	3.98	4.11
Val	4.65 ^‡^	4.59	4.80 ^‡^	5.36	4.51 ^‡^	4.37 ^‡^	4.46 ^‡^	4.71 ^‡^	4.88 ^‡^
TEAA ^4^	35.6	32.3	35.0	36.8	31.7	32.1	32.5	33.6	33.7
MPS ^5^	0.525	0.476	0.516	0.543	0.467	0.473	0.479	0.496	0.497
NEAA^6^ (g/16 g N)									
Ala	6.18	4.81	4.37	5.17	3.85	4.38	4.47	4.71	4.81
Arg	3.17	3.18	5.47	4.70	5.81	3.87	3.93	3.67	3.68
Asp	8.36	8.19	9.77	7.69	9.50	8.45	8.42	8.72	8.47
Glu	11.5	8.62	13.6	17.5	14.7	11.3	11.0	10.6	9.90
Gly	4.56	5.00	4.61	5.21	3.85	4.12	4.13	4.59	4.58
Ser	4.08	3.92	4.86	4.54	4.71	4.05	4.08	4.21	4.14
Tyr	2.98	2.69	3.27	2.88	2.60	2.42	2.47	2.70	2.64
TNEAA	40.8	36.4	46.0	47.7	45.1	38.5	38.5	39.2	38.2
TAA	76.5	68.7	81.0	84.5	76.7	70.6	71.0	72.8	71.9

^1^ MS, Maize silage; RCS, Red clover silage; SBM, Soybean meal; W, Wheat; LS, Lupine seed. ^2^ The TMR was composed of forage and concentrates (0.75:0.25), with targeted ratios of red clover silage (RCS) in TMR of 0.15 (RCS15), 0.30 (RCS30), 0.45 (RCS45), and 0.60 (RCS60) on a DM basis. ^3^ EAA, Essential AA. ^4^ TEAA, Total EAA. ^5^ MPS, Milk protein score calculated as MPS = (g of EAA/kg crude protein)/(g EAA in milk/kg of milk protein) according to Schingoethe [17]. ^6^ NEAA, Non-essential AA. *^,†,^^‡^ Designate the order of the first, second, and third most limiting AAs, respectively, within TMR components or TMR, based on estimations of the AA content of milk protein [18].

**Table 4:** Ruminal degradation (D) in % of crude protein (CP) and amino acids (AAs) of individual feeds and total mixed rations (TMR) after 16 h of incubation.

Item	TMR Components ^1^						TMR ^2^					*p* > F ^3^	
	MS	RC	SBM	W	L	SEM	RCS15	RCS30	RCS45	RCS60	SEM	Linear	Quadratic
D CP ^4^	64.6 ^d^	82.7 ^b^	76.1 ^c^	94.2 ^a^	92.7 ^a^	1.29	75.8 ^c^	80.5 ^b,c^	83.4 ^a,b^	86.3 ^a^	2.60	<0.001	0.49
EAA ^5^													
Cys	61.1 ^d^	75.0 ^c,^*	86.1 ^b,^*	96.0 ^a^	96.0 ^a^	4.32	73.6 ^b^	78.4 ^a^	79.0 ^a^	79.7 ^a,^*	2.20	<0.01	0.09
His	55.0 ^c^*	82.1 ^b^	81.4 ^b^*	94.5 ^a^	95.4 ^a^	2.14	77.0 ^b^	81.3 ^ab^	82.5 ^a^	84.7 ^a^	2.44	<0.001	0.39
Ile	61.5 ^d^	82.2 ^b,c^	77.2 ^c^	86.4 ^a,b,^*	86.3 ^a,^*	2.34	76.5 ^b^	81.2 ^a,b^	83.2 ^a^	84.8 ^a^	2.27	<0.001	0.16
Leu	72.7 ^d,^*	82.4 ^b^	77.6 ^c^	95.9 ^a^	95.7 ^a^	1.26	77.4 ^b^	81.3 ^a,b^	83.0 ^a^	84.5 ^a^	2.42	<0.001	0.29
Lys	26.5 ^c,^*	79.4 ^b^	80.5 ^b,^*	90.4 ^a,^*	93.3 ^a^	1.62	72.3 ^b^	78.1 ^a^	80.3 ^a^	82.3 ^a^	2.17	<0.001	0.08
Met	63.2 ^c^	82.6 ^b^	80.8 ^b,^*	94.4 ^a^	92.9 ^a^	3.11	75.6 ^b^	79.9 ^a,b^	81.5 ^a^	82.9 ^a^	2.52	<0.01	0.23
Phe	65.4 ^c^	81.3 ^b^	78.9 ^b,^*	93.6 ^a^	92.6 ^a^	1.70	76.6 ^b^	80.8 ^a,b^	82.6 ^a^	84.2 ^a^	2.53	<0.001	0.27
Thr	47.3 ^d,^*	81.7 ^b^	78.3 ^c^	91.4 ^a,^*	93.1 ^a^	1.47	73.6 ^c^	78.5 ^b^	81.5 ^a,b^	83.3 ^a^	2.24	<0.001	0.15
Val	67.7 ^d^	83.9 ^b^	76.7 ^c^	92.5 ^a^	92.0 ^a^	1.52	76.6 ^b^	81.6 ^a^	83.8 ^a^	85.3 ^a^	2.17	<0.001	0.10
NEAA ^6^													
Ala	76.3 ^c^	83.7 ^b^	77.8 ^c^	91.9 ^a^	92.7 ^a^	1.40	80.1 ^b^	83.5 ^a,b^	84.7 ^a^	85.8 ^a^	1.82	<0.001	0.19
Arg	38.0 ^d^	76.8 ^c^	82.2 ^b^	93.1 ^a^	96.0 ^a^	2.36	80.9	82.8	84.7	84.9	2.56	0.02	0.46
Asp	42.6 ^d^	86.8 ^b^	81.1 ^c^	91.5 ^a^	93.5 ^a^	1.24	76.3 ^c^	81.9 ^b^	84.6 ^a,b^	87.1 ^a^	2.11	<0.001	0.13
Glu	64.3 ^d^	79.4 ^c^	82.8 ^b^	96.4 ^a^	95.2 ^a^	1.38	79.8 ^b^	83.5 ^a,b^	85.0 ^a^	86.8 ^a^	2.13	<0.001	0.35
Gly	60.9 ^c^	79.8 ^b^	79.4 ^b^	92.7 ^a^	94.4 ^a^	1.37	76.6 ^b^	81.3 ^a^	82.1 ^a^	84.0 ^a^	2.03	<0.001	0.19
Ser	55.0 ^d^	82.2 ^b^	78.8 ^c^	94.4 ^a^	93.7 ^a^	1.38	75.1 ^b^	79.8 ^a,b^	82.0 ^a^	84.2 ^a^	2.39	<0.001	0.28
Tyr	83.6 ^c^	89.0 ^b^	77.5 ^a^	93.4 ^a^	94.4 ^a^	1.12	86.4 ^b^	89.4 ^a^	90.2 ^a^	91.6 ^a^	1.36	<0.001	0.23

^a,b,c,d^ Within a row, LSM with different lowercase letters differ among individual feeds or TMR (*p* < 0.05). * Indicates difference between ruminal degradation of each EAA and degradability of crude protein (D CP) within each individual feed or TMR (*p* < 0.05). ^1^ MS, Maize silage; RCS, Red clover silage; SBM, Soybean meal; W, Wheat; LS, Lupine seed. ^2^ The TMR was composed of forage and concentrates (0.75:0.25), with targeted ratios of red clover silage (RCS) in TMR of 0.15 (RCS15), 0.30 (RCS30), 0.45 (RCS45), and 0.60 (RCS60) on a DM basis. ^3^ Probability of linear or quadratic effect of incrementing proportion of RCS in the TMR. ^4^ D CP, Ruminal degradation of CP after 16 h of incubation and published by Westreicher-Kristen et al. [8]. ^5^ EAA, Essential AA. ^6^ NEAA, Non-essential AA.

**Table 5:** Intestinal digestibility of essential amino acids (AAs) of total mixed rations (TMR) containing increasing proportions of red clover silage.

Item	TMR ^1^					*p* > F ^2^	
	RCS15	RCS30	RCS45	RCS60	SEM	Linear	Quadratic
Intestinal digestibility ^3^							
CP (g/100 g CP)	76.0 ^a^	73.6 ^a^	63.5 ^b^	57.4 ^c^	0.91	<0.001	0.08
Cys	68.3 ^a,^*	68.2 ^a^	53.5 ^a,^*	46.0 ^b,^*	6.39	<0.001	0.41
His	78.1 ^a^	76.1 ^a^	65.4 ^b^	63.1 ^b^	1.36	<0.001	0.92
Ile	70.8 ^c^	80.6 ^a^	73.6 ^b,^*	71.9 ^b,c,^*	1.09	0.94	<0.001
Leu	89.2 ^a,^*	81.0 ^b^	74.0 ^c,^*	72.4 ^c,^*	0.83	<0.001	<0.01
Lys	80.4 ^a^	79.5 ^a^	73.8 ^b,^*	71.7 ^b,^*	0.97	<0.001	0.53
Met	83.4 ^a,^*	84.0 ^a,^*	76.9 ^b,^*	73.4 ^b,^*	2.44	<0.001	0.27
Phe	80.9 ^a^	79.9 ^a^	73.1 ^b,^*	71.5 ^b,^*	0.91	<0.001	0.75
Thr	75.3 ^a^	75.6 ^a^	67.8 ^b^	66.0 ^b,^*	1.07	<0.001	0.34
Val	79.0 ^a^	78.2 ^a^	71.0 ^b,^*	69.3 ^b,^*	0.96	<0.001	0.69
Intestinal absorbable ^4^							
CP (g/100 g)	18.4 ^a^	14.3 ^b^	10.5 ^c^	7.88 ^d^	0.17	<0.001	<0.01
Cys	18.0 ^a^	14.7 ^a,b^	11.3 ^b,c^	9.32 ^c,^*	1.43	<0.001	0.34
His	18.0 ^a^	14.3 ^b^	11.5 ^c^	9.68 ^d,^*	0.24	<0.001	<0.01
Ile	16.3 ^a,^*	15.2 ^b^	12.3 ^c,^*	11.0 ^d,^*	0.22	<0.001	0.57
Leu	20.1 ^a^	15.1 ^b^	12.6 ^c,^*	11.2 ^d,^*	0.14	<0.001	<0.001
Lys	22.3 ^a,^*	17.4 ^b,^*	14.5 ^c,^*	12.7 ^d,^*	0.21	<0.001	<0.001
Met	20.3 ^a,^*	16.9 ^b,^*	14.2 ^c,^*	12.6 ^d,^*	0.47	<0.001	<0.01
Phe	18.9 ^a^	15.4 ^b^	12.7 ^c,^*	11.3 ^d,^*	0.17	<0.001	<0.001
Thr	19.8 ^a^	16.2 ^b^	12.5 ^c,^*	11.0 ^d,^*	0.22	<0.001	<0.01
Val	18.5 ^a^	14.4 ^b^	11.5 ^c^	10.2 ^d,^*	0.17	<0.001	<0.001
Total digestible ^5^							
CP (g/100 g)	94.2 ^b^	94.9 ^a^	93.9 ^b^	94.1 ^b^	0.17	0.22	0.21
Cys	91.6 ^a,^*	93.2 ^a^	90.2 ^a,b,^*	89.1 ^b,^*	1.42	0.03	0.20
His	95.0 ^a,b^	95.5 ^a^	93.9 ^c^	94.3 ^b,c^	0.24	0.01	0.78
Ile	92.8 ^c^	96.3 ^a^	95.6 ^b,^*	95.7 ^a,b,^*	0.22	<0.001	<0.001
Leu	97.6 ^a,^*	96.4 ^b^	95.6 ^c,^*	95.7 ^c,^*	0.14	<0.001	<0.01
Lys	94.6 ^b^	95.5 ^a^	94.9 ^b^	95.0 ^a,b^	0.21	0.53	0.08
Met	96.0 ^a,b,^*	96.8 ^a^	95.7 ^a,b,^*	95.4 ^b^	0.45	0.12	0.14
Phe	95.5 ^b^	96.1 ^a^	95.3 ^b,^*	95.5 ^b^	0.17	0.30	0.23
Thr	93.5 ^b^	94.7 ^a^	94.1 ^a,b^	94.3 ^a^	0.22	0.11	0.05
Val	95.1 ^b^	96.0 ^a^	95.3 ^b,^*	95.5 ^a,b,^*	0.17	0.48	0.08

^a,b,c,d^ Within a row, LSM with different lowercase letters differ among TMR (*p* < 0.05). * Indicates difference between intestinal digestibility, intestinal absorbable and total digestibility of each EAA and of CP within each TMR (*p* < 0.05). ^1^ The TMR was composed of forage and concentrates (0.75:0.25) with targeted ratios of red clover silage (RCS) in TMR of 0.15 (RCS15), 0.30 (RCS30), 0.45 (RCS45), and 0.60 (RCS60) on a DM basis. ^2^ Probability of linear or quadratic effect of incrementing proportion of RCS in the TMR. ^3^ Determined through the mobile-bag technique using the feed residues after 16 h of ruminal incubation. ^4^ Calculated as AA in RUP × ID of AA/100, where IAAA and AA in RUP are in g/kg AA, and ID of AA in percent. ^5^ Calculated as 1000–AA in RUP–IAAA, where TDAA, AA in RUP and IAAA are in g/kg AA.

## Data Availability

The data of this study are available on request from the corresponding author.
